# The Progress of the Biotechnological Production of Class IIa Bacteriocins in Various Cell Factories and Its Future Challenges

**DOI:** 10.3390/ijms25115791

**Published:** 2024-05-26

**Authors:** Yu Wang, Nan Shang, Yueying Huang, Boya Gao, Pinglan Li

**Affiliations:** 1 Key Laboratory of Functional Dairy, College of Food Science and Nutritional Engineering, China Agricultural University, Beijing 100083, China; 2 College of Engineering, China Agricultural University, Beijing 100083, China

**Keywords:** class IIa bacteriocin, cell factories, biotechnological production, food industry application

## Abstract

Class IIa bacteriocins produced in lactic acid bacteria are short cationic peptides with antimicrobial activity. In the search for new biopreservation agents, class IIa bacteriocins are considered to be the best potential candidates, not only due to their large abundance but also because of their high biological activity and excellent thermal stability. However, regulated by the biosynthetic regulatory system, the natural class IIa bacteriocin yield is low, and the extraction process is complicated. The biotechnological production of class IIa bacteriocins in various cell factories has been attempted to improve this situation. In this review, we focus on the application of biotechnological routes for class IIa bacteriocin production. The drawbacks and improvements in the production of class IIa bacteriocins in various cell factories are discussed. Furthermore, we present the main challenge of class IIa bacteriocins, focusing on increasing their production by constructing suitable cell factories. Recombinant bacteriocins have made considerable progress from inclusion body formation, dissolved form and low antibacterial activity to yield recovery. The development of prospective cell factories for the biotechnological production of bacteriocins is still required, which may facilitate the application of bacteriocins in the food industry.

## 1. Introduction

Lactic acid bacteria (LAB) are Gram-positive bacteria that contribute significantly to food biopreservation and have a long-standing history of intimate association with humans [1]. Bacteriocins produced by LAB are ribosomal-synthesised antimicrobial peptides that are capable of inhibiting the survival of other similar or highly related bacterial strains [2]. The significance of LAB bacteriocins in ensuring food safety has been increasingly recognised in recent years due to their proven efficacy as safe, natural antimicrobials for use in food applications [3].

**Figure 1:**
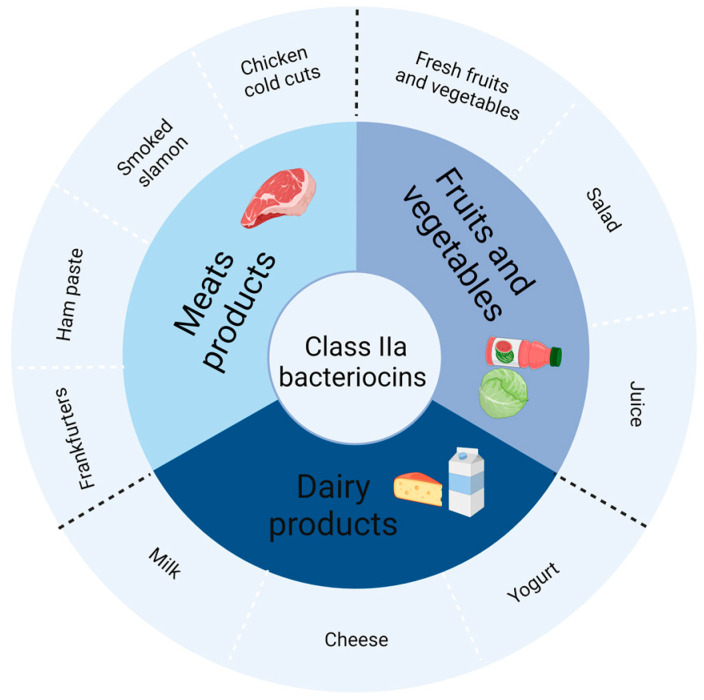
Potential applications of class IIa bacteriocins in the food industry. Created with BioRender.com. accessed on 1 August 2023.

Bacteriocins produced by LAB are commonly divided into three categories based on their genetic and biological properties [1]. Class I is generally regarded as lantibiotics, small peptides (<5 kDa) and has post-translational modification; class II can be subdivided into four categories, which consist of small peptides (with a molecular weight of less than 10 kDa) that exhibit thermal stability. Class IIa (pediocin-like bacteriocins) features a conserved “YGVGN” region in the N-terminal and two cysteine-formed disulphide bridges in the C-terminal. Class IIb bacteriocins, also known as two-peptide bacteriocins, require the coordinated action of two peptides to function fully. Class IIc is circular bacteriocins, and class IId is a miscellaneous group of bacteriocins in addition to the above categories. Generally, it includes some bacteriocins without a leader and some non-pediocin peptides; class III bacteriocins are thermosensitive proteins. Among LAB bacteriocins, nisin, a class I bacteriocin, is the only purified bacteriocin approved for food preservatives and has been used for decades in more than 50 countries. However, the antibacterial activity of nisin is greatly affected by pH. With continued recognition and development, class IIa bacteriocins have emerged as promising agents for food preservatives due to their high antibacterial activity towards foodborne pathogens and essential physicochemical properties, including thermostability and pH stability [4]. Pediocin PA-1, a class IIa bacteriocin, can be commercially used as a crude fermentate in the food industry to reduce *Listeria monocytogenes* [5]. To date, class IIa bacteriocins have been proven to have preservative effects in a broad range of foods, including dairy products, meat, fruit and vegetables (Figure 1) [6].

Class IIa bacteriocins are mainly derived from the fermentation of LAB. However, one fault of current bacteriocin production is the low yield, which limits development and application. Another drawback is the complicated fermentation process, which increases production costs [7]. Although chemical synthesis is a viable strategy, its high cost makes it unsuitable for large-scale industrial production [6]. Techniques for producing recombinant proteins in appropriate cell factories have a long-established history, and biotechnological methods are regarded as “green” and safer than chemical synthesis [8]. Since 1999, researchers have attempted to express class IIa bacteriocins in a heterologous host. With advancements in genetic engineering, many bacteriocins have been produced via biotechnological routes in various cell factories, resulting in advancement achievements (Table 1). However, there are currently no studies that comprehensively summarise the research progress of the biotechnological production of class IIa bacteriocins. This review will concentrate on the advancements in biotechnological pathways utilised for the production of class IIa bacteriocins from 1999 to the present. The limitations and advancements in producing class IIa bacteriocins in different cell factories will also be discussed.

## 2. General Properties of Class IIa Bacteriocins

Class IIa bacteriocins exhibit moderate-to-high heat stability and activity across a broad pH range [36]. Furthermore, they are sensitive to proteases, which renders them no threat to humans [37]. At present, over 30 class IIa bacteriocins have been identified from a variety of LAB, which are the largest and most widely studied class II bacteriocin subgroup [1]. Class IIa bacteriocins have inhibitory activity against various pathogenic strains, including *Bacillus*, *Clostridium*, *Staphylococcus*, etc. The mature peptides of class IIa bacteriocins contain about 36 to 58 amino acids. The N-terminal part is usually charged and hydrophilic and has a conserved YGNGV residue. In addition, the two cysteines at the N-terminal of class IIa bacteriocins are conserved and form at least one disulphide bridge. The C-terminal of class IIa bacteriocins comprises amphiphilic or hydrophobic amino acids. Some class IIa bacteriocins, such as sakacin G, plantaricin 423, pediocin PA-1/AcH, divercin V41 and enterocin A, have an extra disulphide bond in the C-terminal [38]. Studies have shown that bacteriocins containing two disulphide bonds may have higher antimicrobial potency and thermal stability [38,39].

**Figure 2:**
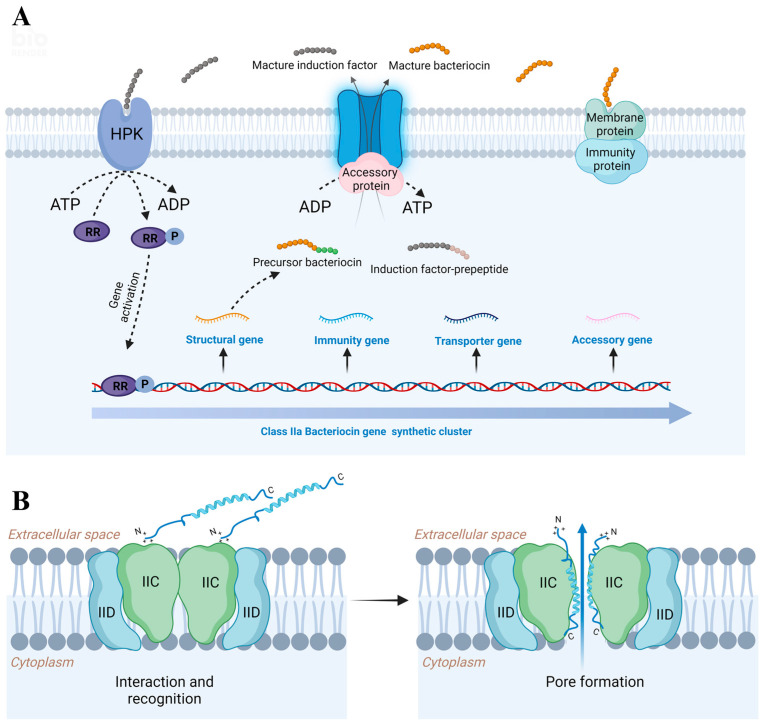
Schematic diagram of synthesis (**A**) and action mechanism (**B**) of class IIa bacteriocins. Created with BioRender.com. accessed on 1 August 2023.

The majority of class IIa bacteriocin genes are encoded on plasmids. The production of class IIa bacteriocins typically requires four genes [4], which are (i) structural genes that encode propeptide bacteriocins; (ii) immune genes encoding immune proteins that confer resistance to bacteriocins; (iii) the ABC transporter gene, encoding the transporter protein and (iv) A genes that encode accessory proteins with unknown functions (Figure 2A). Three-component Quorum Sensing (QS) regulates the synthesis of class IIa bacteriocins [40]. The QS system includes histidine protein kinase (HPK), Induction factor (IF) and Response regulator protein (RR). IF is a signal molecule that senses environmental changes and regulates bacteriocin synthesis. Firstly, IF is synthesised by ribosomes as a precursor peptide, transported to the outside of the cell through the bacteriocin ABC transporter and then cleaved into the active form. Once the active IF reaches a certain concentration threshold, HPK is activated, followed by the phosphorylation of the RR on the intracellular side. The phosphorylated RR then serves as a transcriptional activator to activate the expression of bacteriocin biosynthesis gene clusters (Figure 2A). Class IIa bacteriocins are first synthesised by ribosomes in the form of propeptide consisting of lead peptide and mature bacteriocin, then removed by site-specific proteolytic enzymes during the export process and finally secrete mature bacteriocins into the extracellular environment (Figure 2A). The lead peptide sequence may play a dual role in biosynthesis [38]. First, the presence of the lead peptide makes bacteriocin exist as an inactive form to avoid the damage caused by itself. Second, the lead peptide is a signal that recognises specific transporters and promotes bacteriocin transport. The C-terminus of ABC transporters has a highly conserved ATP binding region, while the N-terminus is a hydrophobic membrane binding region that can cleave the leader peptide at the conserved two cysteines of class IIa bacteriocins. The precursor bacteriocin binds to the hydrolytic domain of the negatively charged ABC transporter through electrostatic interactions. ATP hydrolysis triggers conformational changes in the ABC transporter, which cleaves the twin glycine leader sequence, forming the mature bacteriocin [41]. Nevertheless, it should be noted that not all class IIa bacteriocins are transported via ABC transporters. Due to the difference in the amino acid sequence of the lead peptide, lacking double glycine motifs, such as bacteriocins, including enterin P, bacteriocin 31, and bacteriocin T8 that are exported via a sec-dependent translocation system [42]. The accessory proteins are believed to facilitate the translocation of membranes and assist in processing the leader peptide during mature bacteriocin secretion. Immune proteins are cytoplasmic proteins, and it is generally thought that immune proteins hinder the damage of bacteriocin by directly or indirectly interacting with bacteriocin [41].

It is currently believed that class IIa bacteriocins primarily induce pore formation in the cell membrane of target cells, leading to the leakage of intracellular ions and inorganic phosphate. This disruption of the dynamic balance between the intracellular and extracellular environments affects several essential cellular processes, including replication, transcription and translation, eventually leading to cell death. The mannose phosphotransferase system (Man-PTS), involved in phosphorylation and translocation, serves as the recognition receptor for many class IIa bacteriocins. This system comprises the common PTS protein enzyme I (EI), the phosphor carrier protein histidine-containing protein (HPr) and three sugar-specific proteins consisting of subunits IIA, IIB, IIC and IID. Subunits IIA and IIB are located in the cytoplasm and perform phosphorylation. On the other hand, subunits IIC and IID are transmembrane proteins that facilitate transport [43]. It has been suggested that the extracellular loop of subunit IIC plays a crucial role in the specific target recognition of class IIa bacteriocins [44]. The proposed mechanism of action of class IIa bacteriocins involves an initial interaction between the N-terminal β-sheet domain of the bacteriocin and the extracellular loop of IIC. Subsequently, the α-helical region located in the C-terminus of the bacteriocin is inserted into the membrane to form pores, resulting in solute leakage, the disruption of membrane integrity and ultimately cell death [1,45] (Figure 2B).

## 3. Potential Applications of Class IIa Bacteriocins

Class IIa bacteriocins have attracted widespread attention due to their unique biological activities since their discovery. Researchers have continuously explored and studied the application potential of bacteriocins in the past decades, and bacteriocins have shown great application value in many fields [3,46]. Because bacteriocins can inhibit the growth and reproduction of a variety of foodborne pathogens, they are often added to foods as natural preservatives, such as dairy products, meat products, etc., which not only ensures food safety but also avoids the health risks that chemical preservatives may bring [46]. There are three traditional strategies for the application of bacteriocins in the food industry: purified bacteriocins, fermented products of lactic acid bacteria containing bacteriocins and live bacteria-producing bacteriocins [1]. Since class IIa bacteriocins have a large amount of and broad antibacterial activity, especially strong antibacterial activity against *Listeria monocytogenes*, their physical and chemical properties are relatively stable, showing great potential in food preservation. However, due to the complex approval process for purified bacteriocins for use in food, there are currently no purified class IIa bacteriocins for industrial applications. Nonetheless, several class IIa bacteriocin-producing strains, including *Lactococcus lactis*, *Lactobacillus plantarum*, *Lactobacillus bulgaricus*, etc., have received certification from the U.S. Food and Drug Administration (FDA). As a result, fermentation products of these strains can be sold commercially marketed. Notably, *Lactobacillus* fermentation products containing pediocin PA-1 have found widespread use in the food industry [47,48].

In addition to food biopreservation, bacteriocins also show great potential for application in the medical field [3,49]. Due to the growing problem of antibiotic resistance, scientists are exploring the possibility of using bacteriocins as new antibiotic alternatives. Some bacteriocins have shown activity against multidrug-resistant strains and may be used to treat difficult-to-treat infections. In vivo testing has also demonstrated the effectiveness of bacteriocins in treating infections in animal models. It should be pointed out that although bacteriocins have broad application prospects in the medical field, their practical applications are still limited due to insufficient research on their stability and toxicity, as well as potential side effects and safety in the human body. Thus, the application of bacteriocins in clinical medicine is still in the research stage and has yet to be widely commercialised. Some probiotics can defend against intestinal bacterial pathogens by producing antibacterial substances such as bacteriocins, and their use in treating internal infections is becoming a popular alternative to traditional antibiotics. Therefore, equipping probiotics with the ability to produce heterologous bacteriocins can enhance the intrinsic antimicrobial properties, enabling them to produce antimicrobial substances that target specific pathogens [50]. Geldart et al. achieved the heterologous expression of antimicrobial peptides Enterocin A, Enterocin B and Hiracin JM79 in *E. coli* Nissle 1917. By applying engineered probiotics, they significantly reduced the levels of *Enterococcus faecalis* and *Enterococcus faecium* in mice faeces, demonstrating potential application in the treatment of infections caused by Vancomycin-Resistant *Enterococci* (VRE) [51]. Furthermore, current research shows that Plantaricin Bio-LP1 has an alleviating effect on inflammation caused by multidrug-resistant *E. coli* (MDR-*E. coli*) [52], and Plantaricin BM-1 also has a certain potential in the fight against colorectal cancer [53]. In the future, more applications of class IIa bacteriocins in medicine need to be explored. The heterologous expression of bacteriocins also provides new ideas for the treatment of drug-resistant bacterial infections.

Moreover, in recent years, with the development of nanotechnology and biomaterials science, the application of bacteriocins in novel drug delivery systems and biosensors has also attracted increasing attention [54]. Taking advantage of its antimicrobial and biocompatibility characteristics, scientists have tried to use bacteriocin to construct antimicrobial coatings, drug sustained release carriers and biosensing elements, further broadening its application range in medical and biotechnology fields. In conclusion, class IIa bacteriocins have shown great potential for application in the food field and medical field due to their unique properties, and the development and research of heterologous expression systems for class IIa bacteriocins will be beneficial to us to use this bacteriocin more effectively in the future.

## 4. The Production of Class IIa Bacteriocins by Different Cell Factories

At present, class IIa bacteriocins are mainly obtained through natural biosynthesis [7]. However, due to the influence of the regulatory system, the yield is often relatively low, and the extraction steps are complex, which is not conducive to further research on bacteriocins, such as the antibacterial mechanism, structure–activity relationship and development and application research. A few studies also report that bacteriocins can be synthesised by chemical methods, which involve solid-phase synthesis techniques based on the natural amino acid sequence and active structural characteristics of bacteriocins [6]. However, they are relatively expensive and require more effort to meet the needs of bacteriocins, related research and practical applications. With the in-depth study of the genetic characteristics of class IIa bacteriocins and the widespread application of genetic engineering technology, many scholars have found that producing bacteriocins through heterologous secretion expression can overcome these shortcomings. The study of expressing class IIa bacteriocins can provide crucial evidence for analysing the secretion mechanism of class IIa bacteriocins. It can also offer an effective way for the industrial production and application of class IIa bacteriocins, thus promoting the development of natural biological preservatives. After conducting a thorough analysis of the literature, it has been observed that nearly 20 bacteriocins from lactic acid bacteria have been effectively expressed in over ten different host bacteria strains. These host systems include *Escherichia coli*, lactic acid bacteria, yeast and other organisms. Class IIa bacteriocins subjected to heterologous expression include divercin V41, piscicolin 126, plantaricin 423, pediocin PA-1, enterocin A, enterocin P, etc., as delineated in Table 1. This article focuses on heterologous expression studies of class IIa bacteriocins in *Escherichia coli*, lactic acid bacteria and yeast.

### 4.1. Recombinant Class IIa Bacteriocin Expression Using E. coli as Cell Factories

In order to meet the requirements for the industrial production of bacteriocin, researchers have attempted to construct cell factories suitable for bacteriocin expression in various hosts. *E. coli* is a widely used strain since it is easy to culture, has a concise life and is easily manipulated genetically due to its well-known genetics [55].

Since disulphide bond formation (DSB) is essential for the correct folding of class IIa bacteriocins and the intracellular environment of *E. coli* was unfavourable to the formation of disulphide bonds, the critical problem for class IIa bacteriocin expression in *E. coli* is how to obtain active bacteriocins [56,57]. Richard described a method for expressing divercin V41, a class IIa bacteriocin produced by *Carnobacterium divergens* V41, in *E. coli* (DE3) [9]. In that study, the DvnRV41 peptide was expressed as an activity-translated fusion protein with thioredoxin and was enriched in the cell cytoplasm in a soluble form. After that, Wang et al. combined bacteriocin E50-52 with ubiquitin. They achieved the soluble expression of bacteriocin in the cytoplasm in *E. coli* BL21 (DE3), and the final output of bacteriocin reached 16 mg/L after ubiquitin was removed [16]. Chen et al. fused and expressed bacteriocin NB-C1 and green fluorescent protein (GFP) in the *E. coli* cell-free protein system and finally obtained 2.2 mg/mL soluble from the cytoplasm through continuous cell-free exchange fermentation mode [17]. Ross et al. [11] also adopted this method to achieve the efficient heterologous expression of Plantaricin 423 and Mundticin ST4SA. Thus, GFP-class IIa bacteriocin fusion not only stabilises heterologous expression but also increases yields. However, in the above method, bacteriocins can only be expressed in the cytoplasm of *E. coli*, which is not conducive to the isolation and purification of bacteriocins. Ingham et al. used *E. coli* BL21 (DE3) as a host to fuse piscicolin 126 with a pelB signal peptide and express it under the T7 promoter, finally obtaining the mature secretable form of piscicolin 126 in the supernatant. In addition, divercin AS7 [12], plantaricin Pln1 [15] and pediocin PA-1 [14] were all successfully expressed in the *E. coli* expression system.

The above studies demonstrate that soluble class IIa bacteriocins can be obtained using molecular biology techniques in the *E. coli* expression system. However, the cell wall of *E. coli* limits the secretion of recombinant proteins, making isolation and purification a complex process involving cell lysis, fragmentation, the removal of fusion tags and other challenges. Although bacteriocins can be successfully secreted extracellularly by fusing with pelB signal peptides, the extracellular yields are still meagre, which is unfavourable for industrial production and application. At the same time, *E. coli* is not Generally Recognized as Safe (GRAS) by the FDA and is not permitted for use in the food industry. However, due to its facile genetic manipulation and brief growth cycle, it is a convenient host for genetic engineering studies of bacteriocins.

### 4.2. Recombinant Class IIa Bacteriocin Expression Using LAB as Cell Factories

As opposed to the expression system of *E. coli*, lactic acid bacteria (LAB) have GRAS status, which are an attractive host for expressing heterologous proteins [58]. Moreover, LAB have a stronger ability to exocytose than *E. coli* and synthesise proteins containing disulphide bond formation (DSB) [59]. With the development of bioengineering techniques, more and more researchers are trying to express class IIa bacteriocins in LAB.

Sanchez has described a method for class IIa bacteriocin expression in LAB. In that study, the production and antimicrobial activities of hiracin JM79 produced by *Enterococcus hirae* DCH5 were compared by using *Lactococcus lactis*, *Lactobacillus sakei*, *Enterococcus faecium*, *Enterococcus faecalis* and *Pichia pastoris* as hosts. The study found that the production of HirJM79 by the sec pathway is efficient in LAB. However, the antimicrobial activities of recombinant bacteriocins were lower than native HirJM79 [21]. It is possible that the active expression of bacteriocins will be harmful to the host bacteria. The immunity protein located near the bacteriocin structural gene could protect producer cells from bacteriocin. Based on that, Liu et al. used the co-expression of the structural gene (entP) and immunity gene (entiP) under the control of the constitutive promoter P45 by using the food-grade expression vector pLEB590, a strategy which resulted in enterocin P activity expression in *L. lactis* MG1614 hosts. It achieved 3.9 times more expression than the original host bacteria [20]. In a similar study conducted by Jimenez et al., soluble sakacin A was acquired through co-expression with its cognate immune protein in NZ9000, resulting in higher bacteriocin production and antimicrobial activity compared to the natural producer *Lactobacillus sakei* Lb706 [30].

As a new system for producing antimicrobial peptides, the lactic acid bacteria expression system has been gradually become known and applied, but there are still some problems and shortcomings. Compared with the expression system of *E. coli*, genetic manipulation for lactic acid bacteria is relatively challenging. Consequently, there have been few reports on the heterologous expression of bacteriocin using lactic acid bacteria as the cell factory. Although the problem of bacteriocin secretion can be solved by using LAB as a host, the challenge of low yields due to toxicity to the host still exists. In the future, the genetic engineering of lactic acid bacteria can be further improved to optimise bacteriocin production, for example, by increasing the expression of immune proteins and selecting suitable secreted proteins.

### 4.3. Recombinant Class IIa Bacteriocin Expression Using Yeast as Cell Factories

As a single-celled, eukaryotic organism, yeast exhibits universal culture conditions, rapid growth and reproduction capabilities. Additionally, yeast demonstrates a remarkable tolerance to high hydrostatic pressure. The advantage of the yeast expression system is mainly reflected in the post-translational modification of proteins, such as glycosylation modification. Moreover, a foreign protein expressed by the yeast system can be secreted extracellularly and is easy to purify [60].

There are comparatively few studies regarding the heterologous expression of bacteriocins in yeast. Among these, *Saccharomyces cerevisiae* and *Pichia pastoris* are commonly used as research hosts for bacteriocin production. For example, Schoeman et al. [23] expressed Pediocin PA-1/AcH with *S. cerevisiae*, and although the recombinant yeast showed strong antibacterial activity, the bacteriocin concentration in the supernatant was quite poor. In another study, plantaricin 423 [24] expressed in *S. cerevisiae* exhibited the same situation. It has also been suggested that this may be due to undesirable carbon loss due to the fermentation of *S. cerevisiae* to produce ethanol, further leading to low protein or peptide yields [60]. These unfavourable factors may make *S. cerevisiae* unsuitable as a host for large-scale fermentation to produce bacteriocin. Recently, however, Rossouw et al. successfully achieved Plantaricin 423 and Mundticin ST4SA expressed in *S. cerevisiae* by using the MFα1 secretion signal and codon optimisation. The yields of bacteriocin achieved 18.4 mg/L and 20.9 mg/L, respectively, which were significantly superior compared to the *E. coli* expression system [32].

On the other hand, the ability of *P. pastoris* as a cell factory to produce considerable levels of class IIa bacteriocins for enterocin P [25] and pediocin pa-1/AcH [26] has been demonstrated. Both recombinant bacteriocins were abundantly expressed, except pediocin PA-1/AcH lacked biological activity due to its “collagen-like” nature. Likewise, chimeras of enterocin HF and enterocin CRL35 produced by *P. pastoris* X-33 displayed antimicrobial activity [27]. Furthermore, code optimisation has been performed to produce enterocin A and bacteriocin E 50-52 by *P. pastoris* and *Kluyveromyces lactis* [28]. As a result, the production, antimicrobial activity and specific antimicrobial activity of purified bacteriocin expressed in *Pichia pastoris* are higher than in *K. lactis*. This result is comparable to the study of Enterocin A expression in *P. pastoris* performed by Borrero et al. [29]. Recently, *Saccharomyces boulardii*, which has probiotic functions, such as treating or preventing gastrointestinal diseases, has also successfully expressed leucocin C with activity [31].

Compared with *S. cerevisiae*, *P. pastoris* has a natural advantage in the expression of bacteriocins. The promoter initiation in *P. pastoris* is comparatively facile, and promoter optimisation may be considered to express bacteriocins in the future. In general, *P. pastoris* could be a promising host for recombinant bacteriocin production.

### 4.4. The Other Cell Factories for Recombinant Class IIa Bacteriocins Expression

In addition to studies on the expression of bacteriocins in *E. coli*, LAB and yeast cell factories, researchers have recently tried to obtain class IIa bacteriocins in other hosts. *Corynebacterium glutamicum* is a Gram-positive bacterium with GRAS status. Notably, *C. glutamicum* has become a favourable host cell for the recombinant protein owing to its ability to secrete correctly folded and functional proteins, lack of detectable extracellular hydrolytic enzyme activity and low content of endogenous extracellular proteins. Recently, Goldbeck et al. established *C. glutamicum* as a suitable host for the recombinant production of pediocin PA-1. A synthetic ped ACD operon for pediocin PA-1 that was codon-optimised and cloned under the IPTG-inducible Ptac promoter into pEKEx2. Consequently, the amount of pediocin PA-1 accumulated to approximately 10 μg mL^−1^, and activity achieved 20,480 BU mL^−1^ in bioreactors [33]. Thus, *C. glutamicum* is a suitable strain for pediocin PA-1 production and may also be extended to other class IIa bacteriocins. *Bacillus subtilis*, similar to *C. glutamicum*, exhibits remarkable secretory characteristics and possesses a well-established fermentation process and genetic manipulation techniques. Currently, active bacteriocin expression in *B. subtilis* has also been successfully achieved by Li et al. [34]. In that study, the structural gene encoding mature pediocin PA-1 was connected with vector pHT43 and then introduced into *B. subtilis* WB800N. This cell factory allowed bacteriocin to be expressed without the assistance of the accessory protein and ABC transporter. Interestingly, in addition to efforts to express bacteriocins in microorganisms, researchers have also explored the functional expression of enterocin P in Chinese hamster ovary (CHO) cells, which are widely used as cell factories for producing recombinant biopharmaceutical proteins [35]. As a result, recombinant enterocin P exhibited broad-spectrum antimicrobial properties as native bacteriocin while maintaining stability at high temperatures. Taken together, considering the application prospect of class IIa bacteriocin, more cell factories and strategies could be attempted to improve the expression level and antimicrobial activity.

## 5. Challenges

The pursuit of natural preservatives and the rise in antibiotic-resistant bacteria in the environment have heightened the demand for class IIa bacteriocins, which have been identified as promising antimicrobial agents. Consequently, the necessity for the large-scale production of these bacteriocins has become apparent. The application of bioengineering to enhance bacteriocin production is a promising method for further exploration. In recent years, the development of the biotechnological production of class IIa bacteriocins in diverse cell factories has facilitated the transformation of bacteriocins from inclusion bodies to soluble forms and from low-level expression to recovered production. Nevertheless, the biotechnological production of bacteriocins will still confront a multitude of challenges in the future. While some progress has been made in the heterologous expression of bacteriocins in various cell factories, it has not yet been possible to achieve the industrial-scale production of class IIa bacteriocins. In order to achieve high levels of yield and high activity for bacteriocins, continued efforts are needed to design applicable cell factories and improve the production of bacteriocins through bioengineering. Recent studies have achieved the active expression of class IIa bacteriocins in *Bacillus* sp. expression systems. The genus *Bacillus* is regarded as a safe and well-defined genetic background, which is conducive to the industrial production of target products [61]. The effective development of advanced genetic tools, such as Genome Shuffling and the CRISPR/Cas9 system in *Bacillus*, provides a powerful tool for the further construction of cell factories for the efficient expression of class IIa bacteriocins. Bacteriocins may require a more significant amount to achieve the same effect as chemical preservatives, which also limits their application. According to the hole-forming antibacterial mechanism, the hydrophobicity of the C-terminal and N-terminal of the class IIa bacteriocin can be optimised by site-directed mutagenesis for improving the antibacterial activity of bacteriocins. In addition, genetically engineered bacteriocins must meet stringent safety regulations and specifications set by national regulatory authorities before they can be approved for human consumption. It is worth noting that the approval and safety regulations for new food additives continue to evolve and become more stringent. Thus, there is a lengthy approval process for authorisation for recombination bacteriocins produced by cell factories as new food additives.

## 6. Conclusions

Bacteriocins exhibit considerable potential as natural preservatives in food industries. To date, nisin is the only purified bacteriocin approved for use in food additives. However, several drawbacks of nisin restrict its range of practical applications, e.g., pH strongly influences antibacterial activity. Some class IIa bacteriocins are considered attractive compounds as food preservatives after nisin due to their high antibacterial activity to foodborne pathogenic bacteria (e.g., *Listeria* spp.) and exhibit important physicochemical properties, e.g., thermostability and pH stability. However, the extraction of bacteriocins from their original producing bacteria is difficult and often results in low yields. Thus, considering the growing demand for bacteriocins, further efforts are called upon to improve production efficiency and reduce production costs.

Cell factories for the enhanced production of heterologous products are an established and economically important strategy. After the past few decades, numerous researchers have made efforts to express bacteriocins in different cell factories, and the progress of recombinant bacteriocins has been made from the formation of inclusion bodies, soluble forms and low antimicrobial activity to recovery yields. However, the yields of bacteriocin are still low or only higher than those produced by wild strains that cannot reach industrial production levels.

For the future, there is a pressing need for continued advancements in biotechnology to enhance the production of class IIa bacteriocins. The biotechnological production of class IIa bacteriocins through suitable cell factories would be an economically feasible option and may accelerate their application in the food industry.

## Figures and Tables

**Table 1:** Biotechnological production of class IIa bacteriocins in different cell factories.

Cell Factories	Producing Bacteria	Bacteriocin	Expression Plasmid	Expression Form	Antimicrobial Activity/Indicator Bacteria	Reference
***Escherichia coli***						
*E. coli* Origami (DE3) (pLysS)	*Carnobacterium divergens* V41	divercin V41	pET-32b	intracellular	842.8 AU μg^−1^/*Listeria innocua* F	[9]
*E. coli* AD494 (DE3)	*Carnobacterium piscicola* JG126	piscicolin 126	pET32a	intracellular	247,584 AU mg^−1/^*L. monocytogenes* 4A	[10]
*E. coli* BL21 (DE3)	*Lactobacillus plantarum* 423	plantaricin 423	pRSFDuet-1	intracellular	83.33 BU mL^−1^/*L. monocytogenes* EDG-e	[11]
*E. coli* BL21 (DE3)	*Enterococcus mundtii* ST4SA	mundticin ST4SA	pRSFDuet-1	intracellular	1600 BU mL^−1^/*L. monocytogenes* EDG-e	[11]
*E. coli* Origami (DE3) (pLysS)	*Carnobacterium divergens* AS7	divercin AS7	pET28b+	intracellular	NE	[12]
*E. coli* BL21 (DE3)	*Lactobacillus sakei*	sakacin P	pET28a (+)	intracellular	NE	[13]
*E. coli* TunerTM (DE3)	*Pediococcus acidilactici* LMG2351	pediocin PA-1	pETcoco-2	extracellular	640 BU mL^−1^/*Listeria innocua* DPC3572	[14]
*E. coli* BL21 (DE3)	*L. plantarum 163*	plantaricin Pln1	pET32a	intracellular	NE	[15]
*Escherichia coli* BL21 (DE3)	synthesised	bacteriocin E 50-52	pET SUMO	intracellular	NE	[16]
*E. coli* cell-free system	synthesised	bacteriocin NB-C1	pIVEX 2.4d	intracellular	NE	[17]
***Lactobacillus***						
*Lactobacillus casei* CECT475	*Enterococcus faecium* T136	enterocin A	pSIP411UAI)	extracellular	255,191 BU mg^−1^/*L. monocytogenes* CECT911	[18]
*L. lactis* IL1403	*E. faecium* PLBC21	enterocin A	pMPA15	extracellular	NE	[19]
*L. lactis MG1614*	*E. faecium* LM-2	enterocin P	pLEB590	extracellular	7.24 × 10^4^ AU mg^−1^/*L. monocytogenes* 54002	[20]
*L. lactis* NZ9000	*E. faecium* PLBC21	enterocin A	pMPA15	extracellular	NE	[19]
*L. lactis NZ9000*	*Enterococcus hirae* DCH5	hiracin JM79	pMG36c pNZ8048	extracellular	26,480 BU mg^−1^/*E. faecium* T136	[21]
*L. lactis* NZ9000	*Leuconostoc carnosum* 4010	leucocin C	pLEB690	extracellular	NE	[22]
**Yeast**						
*Saccharomyces cerevisiae* Y294	*P. acidilactici*	pediocin PA-1	YEp352	extracellular	NE	[23]
*S. cerevisiae* L5366h	*L. plantarum* 423	plantaricin 423	YEp352	extracellular	NE	[24]
*P. pastoris* X-33	*E. faecium P13*	enterocin P	pPICZαA	extracellular	10,240 BU mL^−1^/*E. faecium* T136	[25]
*P. pastoris* X-33	*P. acidilactici*	pediocin PA-1	pPICZ*α*A	extracellular	NE	[26]
*P. pastoris* X-33	*Enterococcus hirae* DCH5	hiracin JM79	pPICZαA	extracellular	5217 BU mg^−1^/*E. faecium* T136	[21]
*P. pastoris* X-33	*E. faecium*	enterocin HF and	pPICZαA	extracellular	11,129 BU mL^−1^/*Pediococcus damnosus* CECT4797	[27]
*P. pastoris* X-33	*E. faecium*	enterocin CRL35	pPICZαA	extracellular	2720 BU mL^−1^/*P. damnosus* CECT4797	[27]
*P. pastoris* X-33	*E. faecium* T136;	enterocin A	pPICZαA	extracellular	371,200 BU μg^−1^/*L. monocytogenes* 4031	[28]
*Kluyveromyces. lactis* GG799EA	*E. faecium* T136;	enterocin A	pKLAC2	extracellular	343 BU μg^−1^/*L. monocytogenes* 4031	[28]
*P. pastoris* X-33	*E. faecium NRRL* B-32746	bacteriocin E 50-52	pPICZαA	extracellular	75 BU μg^−1^/*L. seeligeri* 917	[28]
*Kluyveromyces. lactis* GG799EA	*E. faecium NRRL* B-32746	bacteriocin E 50-52	pKLAC2	extracellular	1312 BU μg^−1^/*L. ivanovii* 913	[28]
*P. pastoris* X-33	*E. faecium* T136	enterocin A	pPICZ*α*A; YRC-ALEU2m	extracellular	5528 BU μg^−1^/*L. monocytogenes* CECT	[29]
*K. lactis* GG799EA	*E. faecium* T136	enterocin A	pKLAC2;	extracellular	3252 BU μg^−1^/*L. monocytogenes*	[29]
*H. polymorpha* KL8-1EA	*E. faecium* T136	enterocin A	pBTEA	extracellular	213 BU μg^−1^/*L. monocytogenes*	[29]
*K. lactis* GG799	*Lactobacillus sakei* Lb706	sakacin A	pKLAC2	extracellular	32.5 BU ml^−1^/*E. faecium* T136	[30]
*Saccharomyces boulardii* CNCM I-745	*Leuconostoc carnosum 4010*	leucocin C	pSF-Blast	extracellular	NE	[31]
*Saccharomyces cerevisiae* Y294	*Lactobacillus plantarum 423*	plantaricin 423	yBBH1-MFα1	extracellular	320 AU mL^−1^/*L. monocytogenes* EDG-e	[32]
*S. cerevisiae* Y294	*Enterococcus mundtii ST4SA*	mundticin ST4SA	yBBH1-MFα1	extracellular	533 AU mL^−1^/*L. monocytogenes* EDG-e	[32]
**The other cell factories**						
*Corynebacterium glutamicum* CR099	*Pediococcus acidilactici* 347	pediocin PA-1	pEKEx2	extracellular	20,480 BU mL^−1^/*L. monocytogenes* EGDe:pIMK2	[33]
*Bacillus subtilis* WB800N	*Lactobacillus plantarum* Zhang-LL	pediocin/PapA	pHT43	extracellular	NE	[34]
Chinese hamster ovary cells	*Enterococcus* spp.	enterocin P	pcDNA™3.1(+)	extracellular	NE	[35]

Note: NE: not evaluated in study; AU/mL: arbitrary units per millilitre, the reciprocal of the highest dilution corresponding to the inhibitory indicator strain; BU/mL: one bacteriocin unit, the reciprocal of the highest dilution of the bacteriocin causing 50% growth inhibition.

## Data Availability

Not applicable.
